# The Ætiology and Treatment of Nocturnal Enuresis

**Published:** 1908-05-09

**Authors:** Jas. W. Russell

**Affiliations:** Assistant Physician to the Birmingham General Hospital


### Hospital Clinics.

### THE ÆTIOLOGY AND TREATMENT OF NOCTURNAL ENURESIS.

By JAS. W. RUSSELL, M.A., M.D.(Cantab.), F.R.C.P.(Lond.), Assistant Physician to the Birmingham General Hospital.

In the following series of cases of nocturnal enuresis there are a few points concerning etiology and treatment which appear to be worth recording.

The bearing of sex and age may be passed over very briefly. The sexes are generally said to be equally affected, and the slight preponderance in the female sex to be found in my cases—twenty-five out of forty-five—would probably disappear in a larger series. The disorder dated from infancy in an unusually large proportion of the cases: in 44.4 per cent. it had existed from birth and in another 11.1 per cent. it had first made itself felt in the second or third year. Trousseau states that, though sometimes dating from birth it is generally between the ages of seven and eight that nocturnal enuresis first declares itself.

The rarity of the disorder as met with in my private practice points to its close connection with poverty and depressing conditions of life. Apart from this heredity appears to be by far the most prominent feature in the etiology, a point which is seldom mentioned in the descriptions of the complaint. Of the thirty-one cases in which I have made inquiries on this subject no fewer than fourteen possessed a history of a similar occurrence in some member or members of their family, though occasionally the degree of relation is somewhat remote. The following list gives particulars of the family history in this connection:—

1. Girl, aged 13. Incontinence for three months. Mother suffered for two or three years at the same age, one sister from the age of 5 to 9, and another up to her fifth year.
2. Girl, aged 8. Incontinence from birth. Sister suffers from the same complaint, and mother's brother (now aged 22) from birth.
3. Girl, aged 16. Incontinence from birth. One brother suffered till the age of 14.
4. Boy, aged 21. Incontinence from birth. Three or four of brothers and sisters have been similarly affected.
5. Boy, aged 7. Incontinence a year. Mother suffered "on and off all the time till aged 17 or 18."
6. Girl, aged 11. Incontinence from birth. Eldest sister suffered till aged 10.
7. Girl, aged 16. Incontinence a year. Brother suffered periodically "from boyhood till aged 19."
8. Girl, aged 7. Incontinence five years. Mother suffered "when young."
9. Girl, aged 20. Incontinence from birth. Sister suffered till the age of 22.
10. Boy, aged 22. Incontinence from birth. Uncle suffered up to time of marriage.
11. Boy, aged 5. Incontinence from birth. Sister suffered "for a short time" and father's sister occasionally.
12. Girl, aged 4. Incontinence from birth. Mother suffered in childhood, and still has difficulty in holding urine.
13. Boy, aged 13. Incontinence eleven years. Cousin on father's side has suffered all his lifetime; now aged 23.
14. Girl, aged 12. Incontinence two years. Eldest sister suffered between the ages of 12 and 15 or 16.

It is noticeable that where the family history affects the parents or their relations, this history is to be found five times on the mother's side and only twice on the father's; the relation in one of the latter cases being only that of cousin. In none of the cases had the patient's father been similarly affected, whereas in four the mother had herself previously suffered from incontinence. Possibly, therefore—though the number of cases is too small to establish the point—inheritance of the tendency is especially transmitted on the maternal side. It is to be noticed in this connection that the mothers of two other patients gave a history of a previous attack of chorea.

In other respects the family history has been of but little importance. The mother of one patient suffered from some sort of convulsive attacks in childhood, and in two other cases there was a history of epilepsy in uncles or aunts on the father's side. The uncle of one patient was insane. But none of these patients are to be found in the list of those with a family history of incontinence.

Two patients had themselves passed through an attack of chorea, and one had had epileptic fits, these three being again free from any family history of incontinence. In the last case the enuresis was of eight years' duration, but the fits had taken place for two years only, ceasing a year before the patient came under my notice. The incontinence had occurred practically every night during these two years, and had shown little if any diminution since the cessation of the fits. I could elicit no evidence to suggest that it was the indication of nocturnal attacks of epilepsy, and it seems to have been of too frequent occurrence to admit of this interpretation. The difficulty of distinguishing between genuine incontinence and the discharge of urine during a nocturnal fit is often considerable, and probably a certain number of cases of epilepsy fail of recognition in this manner. Thus, one of my patients, a boy aged twelve, was brought to me for occasional incontinence of urine at night, to which he had been liable since his second year, and which generally occurred for two or three nights in succession, followed by a month's freedom from the complaint. His mother stated that he was mentally dull, but that he was more stupid than usual on the days following an attack of enuresis. The history appeared to me suspicious of nocturnal epilepsy, and I have not included the case in my list.

The fact that only one of the patients had had epileptic fits probably understates the relation of this disorder to enuresis; for it is certain that a considerable number of epileptic patients give a previous history of nocturnal incontinence of urine. Perhaps, also, it is permissible to lay some stress upon the extremely heavy sleep which sometimes accompanies the act, and to compare it with the deep unconsciousness of epilepsy. For example, a young man, aged twenty-one, consulted me for incontinence which had troubled him more or less since birth, with a single interval of two years' duration, when he was about eleven or twelve years of age. With that exception, two or three weeks had been the longest time of freedom from his complaint. There was a very strong family history of consumption: one uncle had suffered from enuresis up to the time of his marriage, and another uncle had had epileptiform fits following an injury. So troublesome was my patient's complaint that he often spent the night on the floor or on a chair, and on those nights never passed urine in his sleep. He declared also that going to bed early served as some protection from the attacks. His sleep was extremely heavy on these occasions, and one night, when sleeping on the floor before the kitchen fire, his clothes caught fire. He did not wake, and told me that, had not his friends perceived the smell of burning and come to his assistance, he would have been badly burned. He had not passed urine, however. There was no other evidence to suggest the occurrence of nocturnal fits, and, on being asked whether, after an attack, he felt any unusual fatigue on waking in the morning, he assured me that the reverse was the case, and that he awoke feeling more tired on the occasions when micturition had been avoided. The occurrence of undue fatigue on waking after the unconscious passage of urine is, in my opinion, an important piece of evidence in favour of nocturnal epilepsy.

There is one further point in ætiology which stands out strongly in my cases—namely, the connection between an attack of some specific fever and the onset or increased severity of the complaint. In three cases the first onset dated from an attack of scarlet fever, and in another the complaint had been greatly aggravated thereby. Two cases dated from an attack of measles, one from "tubercular meningitis" (the patient had well-marked mitral disease), and one from an attack of jaundice. Another patient had been much worse since suffering from "influenza" two months before I saw him. Dr. Still claims an association with rheumatism; but I cannot say more than that one of my patients, as already stated, suffered from mitral disease. In not a single instance was either fright or worms alleged as the cause of the complaint. The latter point, however, is one which I have mostly omitted to inquire for.

One of my patients, a youth, aged twenty-one, stated that his attacks, which dated from infancy, only occurred during the winter, and another patient was reported by her mother to be "a lot worse in winter." I have no further direct evidence as to the influence of cold, if any; but it is noticeable that a large proportion of my cases have first presented themselves for treatment in the six colder months of the year. Thus, taking thirty-nine consecutive cases which have attended on my out-patient days over a a period of exactly three years, the number of first applications are distributed amongst the several months as follows:—

| | | | |
|---|---|---|---|
| January ... | 7 | July ... | 1 |
| February ... | 5 | August ... | 1 |
| March ... | 9 | September ... | 1 |
| April ... | 2 | October ... | 3 |
| May ... | 0 | November ... | 5 |
| June ... | 2 | December ... | 3 |

These figures are somewhat complicated by the fact that my out-patients are all admitted by ticket, and that the year's supply is issued on January 1. Notes are therefore most plentiful in the early part of the year, and become decidedly more difficult to obtain in the last two months. This would not account, however, for the drop in numbers in the spring and summer, whilst it would suggest that the numbers attending in December are probably unduly diminished. On the whole, therefore, I am inclined to think that cold has some effect in increasing the severity of the complaint.

*Treatment.*—I do not propose to restate the well-known facts concerning the treatment of enuresis. Belladonna has not in my hands proved a great success, but I have not cared to push it to any considerable degree under the imperfect conditions of supervision existing in out-patient practice. Strychnine has also failed to effect any marked improvement. Dr. Still has recently recommended the combination of belladonna with the tincture of lycopodium, and also the fluid extract of rhus aromatica; but I have no experience of the use of either of these two drugs.

It has seemed to me that there are a few cases which are favourably influenced by oxide of zinc, if reliance can be placed upon the reports of the patients or their friends. I am, of course, aware of the unsatisfactory nature of such evidence, and I give it only for what it is worth. I am, however, in the habit of giving a trial to this drug in most of my cases of enuresis, and I append brief details of the cases which have seemed to show some improvement during its use. A young man, aged twenty-one, complained of frequent nocturnal micturition dating from birth, but, as a rule, occurring only in winter. On September 8 he was ordered 2 grains of oxide of zinc to be taken three times a day, and this was subsequently changed for a nightly dose. On December 1 he reported that he is "all right so long as he takes the pills; but that if he misses them for a night he is sure to wet the bed." On January 5 he was still attending and still depending upon the drug for freedom from his complaint.

A boy, aged nine, complained of nocturnal incontinence of some months' duration. He was ordered a dose of valerianate of zinc three times a day, and his mother wrote three months later to tell me that his complaint had rapidly ceased, and that there had so far been no return.

A boy, aged twelve, came to me at the hospital some time last autumn suffering from enuresis of two years' standing, for which I prescribed oxide of zinc. On January 12 of the present year he came again, after an interval of a month, having lost his former out-patient paper and ceased attending in consequence. His father stated that his trouble had ceased during the time that he was taking "the pills," but that it had now returned; and he asked for a repetition of the treatment. A week later his mother stated that he was "all right as long as he takes the pills." Up to the end of March there were two single attacks of bed-wetting; but after that time the complaint became again more frequent.

A girl, aged eighteen, had suffered from birth, and passed urine in bed "most nights." A dose of oxide of zinc was ordered at bed time on March 28, and next week she reported that she had had only one attack. On May 9 she had had one attack in the last fortnight.

A boy, aged eight, had had incontinence for six months, occurring every night and sometimes twice in the night. He was first seen during my absence, and was ordered a mixture of bicarbonate of potassium and tincture of hyoscyamus. On October 6 he was no better, and a nightly dose of oxide of zinc was substituted. On October 13 his mother reported that he had passed urine in bed three times during the week, on October 20 once only during the week, and on October 27 he had passed the whole week without a catastrophe.

A girl, aged six, had had incontinence for two or three years, passing urine in bed two or three times every night, and frequently in the daytime also. Oxide of zinc was ordered twice a day, and three weeks later the report was that she had had one attack during the week.

A girl, aged twenty, had suffered since birth, "about twice a fortnight." On January 14 she was ordered a nightly dose of oxide of zinc, and remained free altogether from her trouble up to February 25. On March 18 she reported a recurrence every other night, but had been taking the drug irregularly. A week later she had again become free.

Lastly, a boy, aged nine, had had incontinence since birth night and day, and practically every night. On November 2 he was ordered a grain of oxide of zinc to be taken three times a day, to be increased if necessary up to 3 grains. In the following January a relative reported that the nocturnal enuresis had entirely ceased, but that the incontinence was as bad as ever during the day.

The rest of my cases have shown no improvement under the same treatment.

I am far from claiming that these cases afford any absolute proof of the value of the drug. I can only say, however, that I have not had even this amount of success with other medicines, and that I am inclined to think that it is worth a trial when other measures have failed.

